# Different Levels of Food Restriction Reveal Genotype-Specific Differences in Learning a Visual Discrimination Task

**DOI:** 10.1371/journal.pone.0048703

**Published:** 2012-11-07

**Authors:** Kalina Makowiecki, Geoff Hammond, Jennifer Rodger

**Affiliations:** 1 School of Animal Biology, The University of Western Australia, Crawley, Western Australia, Australia; 2 School of Psychology, The University of Western Australia, Crawley, Western Australia, Australia; Université Pierre et Marie Curie, France

## Abstract

In behavioural experiments, motivation to learn can be achieved using food rewards as positive reinforcement in food-restricted animals. Previous studies reduce animal weights to 80–90% of free-feeding body weight as the criterion for food restriction. However, effects of different degrees of food restriction on task performance have not been assessed. We compared learning task performance in mice food-restricted to 80 or 90% body weight (BW). We used adult wildtype (WT; C57Bl/6j) and knockout (ephrin-A2^−/−^) mice, previously shown to have a reverse learning deficit. Mice were trained in a two-choice visual discrimination task with food reward as positive reinforcement. When mice reached criterion for one visual stimulus (80% correct in three consecutive 10 trial sets) they began the reverse learning phase, where the rewarded stimulus was switched to the previously incorrect stimulus. For the initial learning and reverse phase of the task, mice at 90%BW took almost twice as many trials to reach criterion as mice at 80%BW. Furthermore, WT 80 and 90%BW groups significantly differed in percentage correct responses and learning strategy in the reverse learning phase, whereas no differences between weight restriction groups were observed in ephrin-A2^−/−^ mice. Most importantly, genotype-specific differences in reverse learning strategy were only detected in the 80%BW groups. Our results indicate that increased food restriction not only results in better performance and a shorter training period, but may also be necessary for revealing behavioural differences between experimental groups. This has important ethical and animal welfare implications when deciding extent of diet restriction in behavioural studies.

## Introduction

Analysis of rodent learning behaviour is used in drawing conclusions regarding gene pathways responsible for specific behaviours, in development of pharmaceutical products and in use of transgenic mice as models for human diseases [1]. Given these uses, controlling potentially confounding factors, such as motivation, is important. However, there are acknowledged difficulties in training mice to learn even simple tasks, and inducing a biological stressor is necessary to motivate learning. The Australian code of conduct for the care and use of animals for scientific purposes states:

*“Positive reinforcement is the preferred method to motivate an animal to modify its behaviour or to perform specific tasks. However, in some cases the inducement may need to be some form of biological stress, in which case, it must be as mild as possible. Severe deprivation of water, food, social interaction or sensory stimuli must not be used.”* [2].

This raises the question of what level of food restriction is appropriate to motivate the animal sufficiently to ensure task learning, without compromising health or welfare.

A review of the literature reveals that most rodent (rat and mouse) behavioural experiments reduce animal weights to between 80 and 90% of free-feeding body weight (BW) without adverse effects. However, different weight reduction levels between experiments likely affects stress and motivation of the animals, potentially confounding results and making replication or comparisons between studies problematic. Such variation in learning performance has been shown in experiments comparing food and water restriction as motivators [3], and is evident when comparing punishment and negative reinforcement [4], [5], [6], [7]. In particular, the degree of motivation may have greater influence when measuring subtle behavioural differences, which are of particular interest in studies using mouse models to examine genetic pathways in complex behaviours. Despite these concerns, the effect of different food restriction levels on learning and reverse learning performance in mice has not been assessed.

Our previous work demonstrated a subtle learning deficit in ephrin-A2^−/−^ knockout mice [8]. These mice do not express ephrin-A2, a cell surface protein involved in guiding axons to form topographically organised connections in the brain [9], [10]. Despite mild topographic abnormalities in the visual system [11], ephrin-A2^−/−^ mice have normal visual acuity, visual behaviour and overall activity levels, making them suitable models for visual behavioural experiments [10], [12]. When food restricted to reduce weight to 80% ephrin-A2^−/−^ mice performed normally on a two-choice visual discrimination task, but when measuring bias for repeating or switching from the previous response (learning strategy) during the early phases of reversal learning, adopted a suboptimal neutral strategy [11]. Here we food restricted WT and ephrin-A2^−/−^ mice to 80 or 90% of their original free-feeding weight and compared the impact of different degrees of weight restriction (i.e. different motivation level) on initial and reversal learning task performance in a two-choice visual discrimination task.

## Materials and Methods

### Ethics Statement

All procedures in this study were in accordance with NIH guidelines, The institutional ethics committee (University of Western Australia Animal Ethics Committee) specifically approved this study.

### Subjects and Housing

The study used ten WT (C57Bl/6J strain, two males) mice and ten ephrin-A2^−/−^ mice (three males; ANOVAs confirmed no significant differences between sexes on any dependent variable (all *p*s >.05). WT mice were purchased from Animal Research Centre (Murdoch University). Ephrin-A2^−/−^ mice were originally created by Feldheim and colleagues (2000) to carry a homozygous null mutation of the ephrin-A2 gene. Ephrin-A2^−/−^ mice were bred from heterozygous parents at the Biomedical Research Facility, The University of Western Australia, and were backcrossed for >10 generations on a C5Bl/6J background. Mice were genotyped at weaning, as described previously [8]. Mice were age matched, aged 8–10 weeks old when commencing the experiment.

Mice were housed in standard cages (45 cm×29 cm×12 cm) in groups of 2–3, separated by sex and genotype. Mice were kept in controlled environmental conditions (temperature 22°±2°C, relative humidity 50% ±10%) on a 12-hr light-dark cycle (lights on 7 a.m.–7 p.m.). Mice were habituated to the experimenter and to the maze 2–5 days prior to commencing the visual discrimination task. Each mouse was handled for approximately 30 minutes, over two 15-minute sessions, each day.

### Dietary Restriction

Mice in the different weight restriction groups were trained in separate cohorts. To facilitate learning, diet restriction began two days prior to commencing training. This aimed to reduce mice to 80% or 90% of their free-feeding body weight. Mice were weighed daily and food intake adjusted using a daily-based controlled diet to reach and maintain target body weights and ensure animals remained healthy. Water was available *ad libitum* throughout the experiment.

### Visual Discrimination Task

Mice completed a visual discrimination task in two phases. Mice were initially rewarded for one stimulus (‘learning phase’). After mice reached criterion (defined as at least 80% correct for three consecutive sets of 10 trials), the rewarded stimulus was switched to the opposite, previously incorrect stimulus (‘reverse phase’). Mice were terminally anesthetised 24 hours after reaching criterion performance in the reverse task or after 35 days without reaching criterion.

### Apparatus and Procedure

The visual discrimination task was carried out using a Y-maze, fitted into a 50 cm^2^ box, with visual stimuli at each end of the Y-maze arms (25 cm long). Stimuli consisted of two 5 cm^2^ laminated black and white striped cards at.37 cycles per degree. Both genotypes are capable of distinguishing this spatial frequency [10]. Stimuli in either maze arm were identical except for orientation; one maze arm displayed the horizontal stimulus and the other, vertical. Position of horizontal and vertical stimuli (left vs. right maze arm) followed a random schedule, with the constraint of equal number of trials in right and left arms. The trial schedule changed each day, repeating every seven days. Rotation, rather than transferring laminated cards between arms, ensured mice did not learn an olfactory cue associated with the card instead of the stripe orientation stimulus. The same motion, as if rotating stimuli, was made when correct stimulus position remained the same as the previous trial so that mice did not learn a position change cue (experimenter’s movement and noise of Velcro used to attach stimuli to the maze wall) rather than discriminating between visual stimuli. It also ensured time between trials remained similar whether there was a position switch or not. Random allocation determined which stimulus was rewarded in the learning phase, with the constraint half the mice in each genotype received rewards for the horizontal and half for vertical. The rewarded stimulus was also counter-balanced across cage groups (i.e. mice housed together were rewarded for opposite stimuli) and sex.

Mice were placed at the start of the Y-maze, nose aligned to a centre mark. To receive a reward, mice had to proceed at least halfway down the maze arm containing the correct stimulus (referred to as a correct response). While still in the maze arm, immediately after approaching the correct stimulus mice were rewarded with peanut butter offered as a thin coating on a wooden skewer. Mice were allowed to eat for 2 seconds. Mice were not punished for approaching the incorrect stimulus (referred to as an incorrect response). If mice did not approach a stimulus after 1 minute on the first day, and after 30 seconds every day thereafter, the trial was deemed a non-response (included in analyses as an incorrect response). Mice restricted to 80%BW completed up to 40 trials per day, whereas mice restricted to 90%BW completed 30 trials per day as they failed to reliably perform the task after this number. To adjust for this variation, data were analysed by number of trials completed. However, similar results were obtained when data were analysed by number of days to criterion (data not shown).

The reverse phase commenced the day after mice reached criterion performance. In the reverse phase the opposite, previously incorrect stimulus was rewarded. All other aspects of the reverse task were identical to the initial learning phase.

### Measures

Performance in the Y-maze visual discrimination task was measured as number of trials to criterion, accuracy (percentage correct responses) and learning strategy index. To quantify bias towards repeating or switching from the previous response, learning strategy index was calculated. This was calculated as probability of an incorrect repeat (r) when switch response is required (s): P(r/s) subtracting probability of making incorrect switch when a repeat is required: P(s/r) [11].
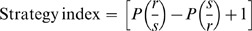



Learning strategy index quantifies bias on a continuum between win-repeat (selecting the same rewarded stimulus) and win-switch (changing to the unrewarded stimulus after a reward). Scoring.5 indicates a neutral strategy (repeating as often as switching), >.5 indicates tendency towards win-repeat strategy and <.5, tendency towards win-switch strategy [13].

## Results

### Weight Restriction Affects Number of Trials to Criterion

Survival analysis (Mantel-Cox) was conducted to assess whether weight restriction groups or genotypes differed in number of trials to reach criterion performance.

**Figure 1:**
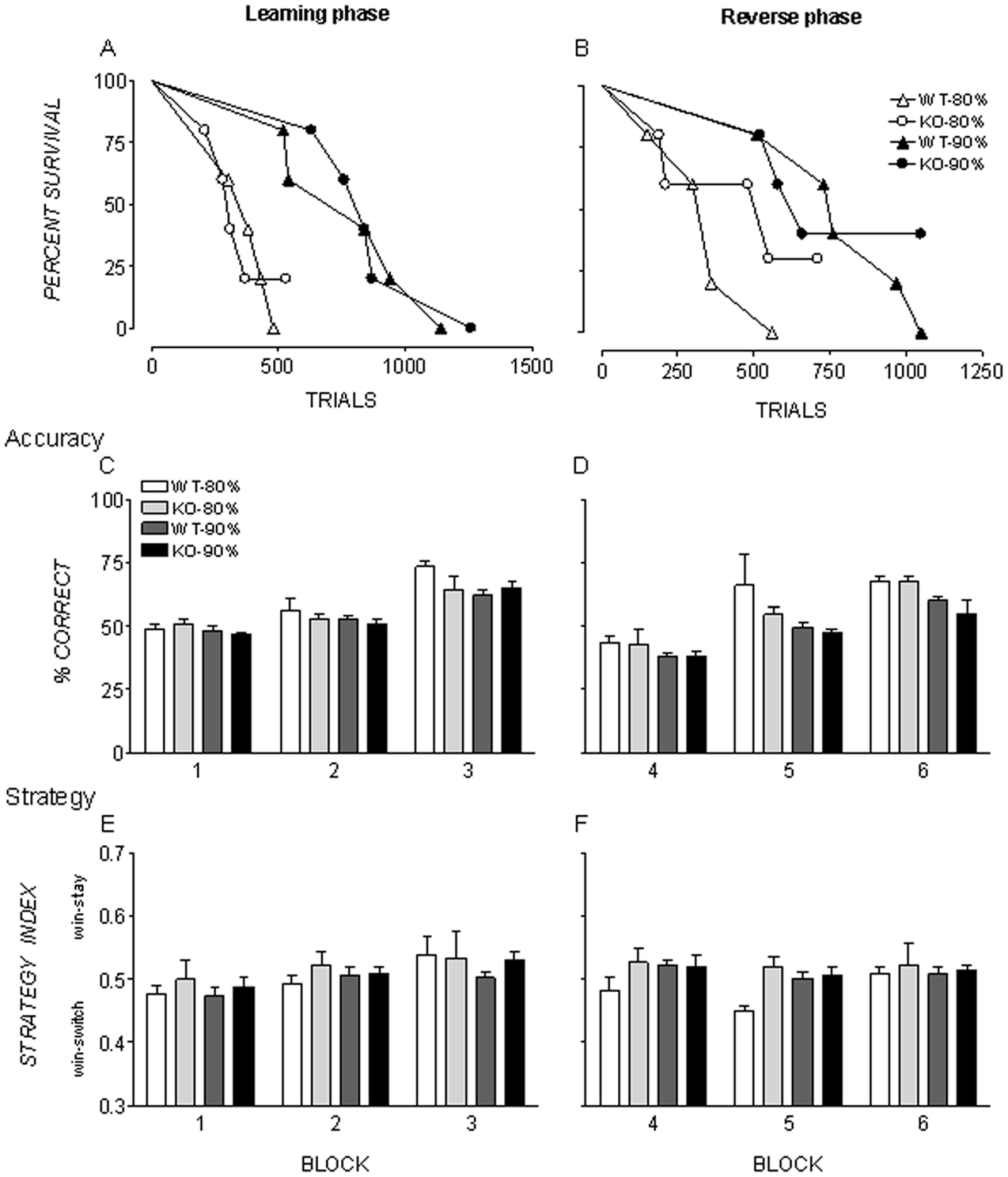
Survival analysis, mean accuracy scores and strategy index scores for each block. Survival analysis is shown as percentage of subjects remaining in the learning phase (A) and the reverse phase (B) as a function of number of trials completed. Accuracy is shown as percentage correct Genotypes (ephrinA2−/knockout  =  KO; wildtype  =  WT) and body weight restriction groups (90% and 80% of free-feeding weight) are represented separately. Error bars represent the standard error of the mean.

Survival analysis showed the 90%BW group had significantly more trials to reach criterion in the learning phase (Median: 839.5 trials) than the 80%BW group (Median: 339.5 trials), χ^2^ (1) = 15.80, *p*<.001 (Figure 1A). In the reverse phase (Figure 1B), the 90%BW group took significantly more trials than the 80%BW group to reach criterion (Median: 90%BW = 745 trials; 80%BW = 360), χ^2^ (1) = 7.592, *p* = .01.

Within weight restriction groups, genotypes did not differ significantly in number of trials to reach criterion performance in the learning or reverse phase. In the learning phase, genotypes required a similar number of trials in the 80%BW group, (Median: ephrin-A2^−/−^ = 310, WT = 380 trials), χ^2^ (1) = .05, *p = *.82; and in the 90%BW group, (Median: ephrin-A2^−/−^ = 839, WT = 840 trials), χ^2^ (1) = 0.11, *p = *.74. One KO mouse in the 80%BW group failed to reach criterion and was excluded from analysis. Likewise, in the reverse phase genotypes did not differ significantly in number of trials to criterion in either the 80%BW group (Median: ephrin-A2^−/−^ = 550, WT = 360 trials), χ^2^ (1) = 7.11, *p = *.40; nor in the 90%BW group, (Median: ephrin-A2^−/−^ = 660, WT = 760 trials), χ^2^ (1) = 0.41, *p = *.52. Four ephrin-A2^−/−^ mice (two from each weight restriction group) failed to reach criterion and were excluded from the reverse phase survival analysis.

### Weight Restriction Affects Reverse Learning Accuracy and Strategy in a Genotype-dependent Fashion

Inferential statistics to analyse whether genotypes and weight restriction groups differed in percentage correct scores and learning strategy index over time, using trials as a repeated measure in a mixed ANOVA, were deemed inappropriate due to reducing sample size over time (in a non-random manner) when mice reached criterion performance. To overcome this problem, for each mouse the total number of trials to criterion was divided evenly into three blocks for both phases: the initial learning period (block 1), the middle learning period (block 2), and the end learning period - the final third of trials before reaching criterion (block 3). Likewise, in the reverse phase total trials for each mouse were divided in the same manner (block 4, block 5 and block 6, respectively). Each block included one third of total trials, with means calculated for each block. The learning phase and reverse phase were analysed separately using two-way mixed ANOVAs with block as the within-subjects factor and weight restriction group (80%, 90%) and genotype (WT, ephrin-A2^−/−^) as the between-subjects factors.

### Accuracy

As presented in Figure 1C, in the learning phase mice in all groups scored close to chance (50%) in block 1 and block 2, while scores increased in block 3.

A mixed two-way ANOVA on percentage correct scores in the learning phase showed scores significantly increased between blocks, *F* (2, 32) = 67.76, *p<*.001, η_p_^2^ = .81. There was no significant main effect of weight restriction percentage, *F*(1, 16) = 3.25, *p = *.09, η_p_^2^ = .17, or genotype, *F*(1, 16) = 1.00, *p = *.33, η_p_^2^ = .06. There were no significant interactions (all *p*s >.05).

In the reverse phase (Figure 1D), percentage correct followed a similar pattern to the initial learning phase. Though means were similar, the 80%BW group scored higher in all blocks, with a more pronounced difference between the 80%BW and 90%BW groups in block 6. In block 5 the WT 80%BW mean was higher than all other groups.

A mixed two-way ANOVA on percentage correct scores in the reverse learning phase showed significant differences between blocks (within-subjects factor), as scores significantly increased between blocks, *F* (2, 32) = 18.60, *p<*.001, η_p_^2^ = .54. There was a significant main effect of weight restriction group, *F*(1, 16) = 19.71, *p = *<.001, η_p_^2^ = .55, but no main effect of genotype, *F*(1, 16) = 2.29, *p = *.15, η_p_^2^ = .13, and no significant interactions (all *p*s *>*.05). Follow up tests (Tukey HSD) showed WTs at 80%BW differed significantly to 90%BW mice in both genotypes, (WT: *p = *.02; ephrin-A2^−/−^ : *p = *.003) but there were no other significant differences between groups (all *p*s >.05).

### Learning Strategy

As shown in Figure 1E, in the initial learning phase, learning strategy scores for all groups remained close to neutral (.5), denoting equal probability of switching as repeating the response of the previous trial, increasing slightly by block 3. Mean strategy index changed significantly between blocks, *F* (2, 32) = 3.32, *p = *.049, η_p_^2^ = .17. There was no significant main effect of weight restriction group, *F*(1, 16) = 0.76, *p = *.40, η_p_^2^ = .05, or genotype, *F*(1, 16) = 2.11, *p = *.17, η_p_^2^ = .12, and no significant interactions (all *p*s *>*.05).

In the reverse learning phase (Figure 1F), there were greater mean differences in learning strategy index scores between WT mice at 80%BW compared to other groups in block 4 and block 5. WT mice at 80%BW tended towards ‘win-stay’ strategy (denoted by score >.5), while ephrin-A2^−/−^ mice at 80%BW and both genotypes in the 90%BW group showed a neutral strategy throughout all blocks in the reverse phase (scores close to.5). The strategy differences decreased with successive blocks, with all groups showing a neutral strategy in block 6.

Block 6 was excluded from the analysis as it violated the homogeneity of variance assumption and no differences between groups were hypothesised in this block [11]. A mixed two-way ANOVA on learning strategy index on blocks 4 and 5 in the reverse learning phase showed no significant difference between blocks (within-subjects factor), *F* (1, 16) = 2.04, *p = *.17, η_p_^2^ = .11. There was no significant main effect of weight restriction group, *F*(1, 16) = 2.67, *p = *.12, η_p_^2^ = .14, but there was a significant main effect of genotype, *F*(1, 16) = 7.63, *p = *.01, η_p_^2^ = .32, and a significant interaction between weight restriction group and genotype, *F*(1, 16) = 6.55, *p = *.02, η_p_^2^ = .29. Follow up tests (Tukey HSD) showed WTs at 80%BW differed significantly to ephrin-A2^−/−^ mice at 80%BW (*p* = .01) and to both genotypes of 90%BW mice, (WT: *p* = .04; ephrin-A2^−/−^: *p* = .03) but there were no significant differences between any other groups.

**Figure 2:**
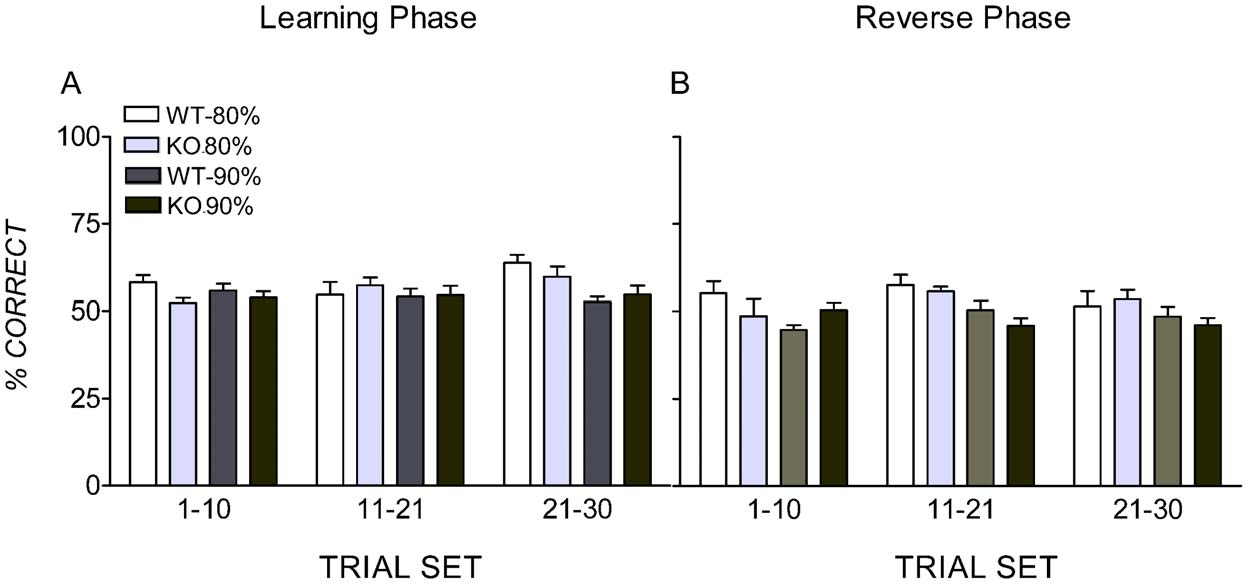
Mean accuracy scores for sets of 10 trials. Results are shown separately for the learning (A) and reverse phases (B). Genotypes (ephrinA2−/knockout  =  KO; wildtype  =  WT) and body weight restriction groups (90% and 80% of free-feeding weight) are represented separately. Error bars represent the standard error of the mean.

To examine whether differences between weight restriction groups observed in the other measures were due to 90%BW mice becoming satiated in later trials within each day, trials were analysed in sets of 10. Percentage correct was calculated for the first, second and third set of 10 trials each day and means calculated from all days to criterion in the learning phase (Figure 2A) and reverse phase (Figure 2B). Repeated-measures ANOVAs showed subjects did not change significantly between 10 trial sets in the learning phase, *F*(2, 38) = 2.57, *p = *.09, η_p_^2^ = .12, nor in the reverse phase, *F*(2, 38) = 1.46, *p = *.25, η_p_^2^ = .07.

## Discussion

### Summary

Our results suggest that differences in weight restriction affect performance in a visual discrimination learning task. Regardless of genotype, 90%BW mice required more than twice as many trials to acquire the initial task compared to 80%BW. In addition, the more severe food restriction condition revealed genotype-specific differences in reverse learning accuracy which were not present when mice were tested at 90%BW.

### Role of Stressor Severity in Task Performance

Our study comparing different levels of food restriction complements other studies indicating that learning rates are dependent on the type of stressor. For example, water-restricted mice acquired an operant learning task roughly 5 days earlier than food-restricted mice (90–85% free-feeding weight) [8], [11]. In addition, learning rates increase when using punishment or negative reinforcement: with punishment (air puffs), mice learnt a Y-maze visual-tactile discrimination in 4 days [6]; in a swim equivalent of the Y-maze (negative reinforcement), C57Bl/6J mice learnt the task in 3–4 days [3], compared to 14 days for mice food restricted to 80%BW in the present study. Interestingly, combining multiple stressor types may be additive, as electric shock further accelerated learning in rats in a directional water Y-maze test [4], [5]. Our results add that learning outcomes are affected not only by stressor type (as discussed above) but also by stressor severity.

The extent and type of food restriction has previously been shown to impact on performance by directly affecting learning and/or by altering drive. Caloric restriction generally has beneficial effects on brain function [14] and previous studies have shown that chronic food restriction (to 82–85%BW) can directly improve memory [15], potentially by upregulating brain derived neurotrophic factor (BDNF) expression and increasing neurogenesis [16]. However, there is also evidence that the severity of food restriction may affect performance rather than learning *per se*; both restricted and non-restricted mice acquired discrimination between odours, but only expressed this discrimination when food restricted [17]. Nonetheless, food restricted mice showed improvements in learning even when changes in drive were controlled [15]. Additional experiments controlling for these factors would be necessary to determine the relative impact of different degrees of food restriction on learning and performance in our study.

### Ephrin-A2^−/−^ Mice Show Normal Learning, but Abnormal Reverse Learning that is Dependent on Weight Restriction Level

Within weight restriction groups, genotypes did not differ in time to criterion, accuracy or strategy in any testing block, confirming previous findings that ephrin-A2^−/−^ mice do not differ to WT mice in learning ability or memory in acquisition of a visual discrimination task, as previously reported [13]. However, we found a significant difference in reverse learning accuracy and strategy between WT and ephrin-A2^−/−^ mice that was evident only under sufficient levels of food restriction. WT mice restricted to 80% BW showed significantly higher accuracy compared to either genotype at 90%BW, accompanied by a tendency towards a win-switch strategy, which differed significantly from the neutral strategy adopted by both genotypes at 90%BW and by ephrin-A2^−/−^ at 80%BW. The higher accuracy accompanying the win-switch strategy suggests that this strategy is advantageous when reward contingencies are uncertain. This also complements previous reports using a Bayesian optimal-observer analysis, which also suggested that a neutral strategy in the early phases of reverse learning is sub-optimal [8]. The reverse learning strategy difference between weight restriction groups in WTs, but not in ephrin-A2^−/−^ mice may reflect differences in the ability of ephrin-A2^−/−^ mice to attend to specific visual features and/or their reward value.

### Ethical Considerations

Although the differences in motivation we describe might suggest that mice weight restricted to 80%BW experience greater stress than mice restricted to 90%BW, evidence indicates restricted food access (either in laboratories or in the wild), even in the long term, is not unusual or undesirable. There is research consensus most species will become obese if allowed free access to food and experience health and longevity benefits if food restricted to some degree [18], [19]. Dietary restriction has been taken further than the 80% described here: rats reduced to 75% of their free-feeding weights lived longer than ad libitum-fed control rats without any adverse health consequences [20]. Although compared to ad-libitum fed controls rats restricted to 85%BW had higher corticosterone levels overall, the difference was not significant between groups at any of the 5 weekly measures [21]. Another study found no difference in corticosterone levels between food restricted and ad libitum-fed rats [22]. When exposed to restraint stress, calorie restricted rats had significantly lower corticosterone levels than ad libitum-fed rats, suggesting lowered stress reactivity with calorie restriction [23]. Furthermore, adrenal glands were larger in ad-libitum fed controls than rats restricted to 85%BW [24]. These results suggest that food restriction up to 25% loss of body weight does not adversely affect animal welfare [22]. Furthermore, the resulting higher motivation significantly reduces duration of experimental procedures. Taken together, the welfare and cost benefits favour use of more severe weight reduction to motivate mice when using food rewards as reinforcement in a learning task.

### Conclusion

In summary, we show that the severity of food deprivation is a key parameter in the design of behavioural studies. Changes in motivation due to hunger are a major confounding factor in behavioural studies and may result in failure to detect differences between experimental groups. Further investigation of genotype-specific differences in learning behaviour under different types and severity of stressors may provide valuable insights into the specific reward pathways underpinning learning and reward value appraisal.
